# Chemistry

**Published:** 1863

**Authors:** 


CHEMISTRY: By WILLIAM THOMAS BRANDE, D.C.L., F.R.S.L. & E. of Her Majesty's Mint, Member of the Senate of the University of London, and Honorary-Professor of Chemistry in the Royal Institution of Great Britain; and ALFRED SWAINE TAYLOR, M.D., F.R.S., Fellow of the Royal College of Physicians of London, and Professor of Chemistry and Medical Jurisprudence in Guy's Hospital. PHILADELPHIA: BLANCHARD & LEA. 1863.

This is a full-sized octavo volume of 696 pages, closely printed, but in fair type. From the hasty examination we have been able to give it, we think it will be found to be one of the best text-books for students that have been published, and also a good work for reference for the general practitioner. The following, from the preface, will give an idea of the general character of the work:—

"In addition to the general properties of bodies, we have, attached to the description of each substance, a summary of its most important characters, with an account of the special tests required for its detection. The student will thus have in this book a manual of practical chemistry. As an adjuvant to this branch of the science, the subject of practical toxicology has been introduced in reference to the most important *poisons*, and the processes for their detection. We have also treated, as fully as our space would permit, the chemical principles on which *photography* is based, and have given some practical rules for the guidance of those who wish to apply their chemical knowledge to this interesting art."

For sale by W. B. Keen & Co., Booksellers in this city.

